# Adapting prenatal iron supplementation to maternal needs results in optimal child neurodevelopment: a follow-up of the ECLIPSES Study

**DOI:** 10.1186/s12884-022-05033-y

**Published:** 2022-09-17

**Authors:** Lucía Iglesias-Vázquez, Carmen Hernández-Martínez, Núria Voltas, Josefa Canals, Pilar Coronel, Mercedes Gimeno, Victoria Arija

**Affiliations:** 1 Nutrition and Mental Health (NUTRISAM) Research Group, Universitat Rovira I Virgili, 43204 Reus, Spain; 2 Institut d’Investigació Sanitaria Pere Virgili (IISPV), 43204 Reus, Spain; 3 Department of Psychology, Research Centre for Behavioral Assessment (CRAMC), Faculty of Education Sciences and Psychology, Universitat Rovira I Virgili, 43007 Tarragona, Spain; 4 Department of Psychology, Faculty of Education Sciences and Psychology, Serra Húnter Fellow, Universitat Rovira I Virgili, 43007 Tarragona, Spain; 5 Meiji Pharma SpainES (Formerly Tedec-Meiji Farma S.A, 228802 Alcalá de Henares, Madrid Spain; 6 Collaborative Research Group On Lifestyles, Nutrition, and Smoking (CENIT). Tarragona-Reus Research Support Unit, IDIAP Jordi Gol, 43003 Tarragona, Spain

**Keywords:** Iron supplementation, Prenatal, Neurodevelopment, Cognitive development, Language development, Motor development

## Abstract

**Background:**

Prenatal prescription of standard iron supplements to prevent iron deficiency appears not to be appropriate for all women and their children, as some women may be at risk of iron deficiency and others at risk of iron excess early in pregnancy. The present study aimed to assess whether prenatal iron supplementation adapted to the needs of each pregnant woman affects their child’s neurodevelopment.

**Methods:**

Follow-up of a community-based RCT involving 503 mother–child pairs. Non-anaemic pregnant women recruited in Tarragona (Spain) early in pregnancy were prescribed a daily iron dose based on their initial haemoglobin levels: *Stratum* 1 (Hb = 110–130 g/L, 80 or 40 mg/d of iron) and *Stratum* 2 (Hb > 130 g/L, 40 or 20 mg/d of iron). Women receiving 40 mg/d were considered the control group in each *Strata*. The child’s neurodevelopment was assessed at 40 days of age using the Bayley Scales of Infant Development-III (BSID-III). Adjusted multiple regression models were used.

**Results:**

Multiple regression analyses showed no association between the intervention and control group within each *Strata* on the BSID-III scores on any of the developmental scales in children, including cognitive, language, and motor development: *Stratum* 1 (β 1.46, 95%CI -2.15, 5.07; β 1.30, 95%CI -1.99, 4.59; and β 2.04, 95%CI -3.88, 7.96, respectively) and *Stratum* 2 (β -4.04, 95%CI -7.27, 0.80; β -0.36, 95%CI -3.47, 2.75; and β -3.76, 95%CI -9.30, 1.78, respectively).

**Conclusions:**

In non-anaemic women in early pregnancy, no differences were found in the cognitive, language and motor development of children at 40 days of age between the dose of iron tested in each case –adjusted to initial Hb levels– compared to the dose of the control group. Further studies are guaranteed to confirm our findings.

**Trial registration:**

The ECLIPSES study was registered at www.clinicaltrialsregister.eu as EudraCT number 2012–005,480-28.

**Supplementary Information:**

The online version contains supplementary material available at 10.1186/s12884-022-05033-y.

## Background

Accumulating evidence indicates that maternal iron status during the gestational period is of great importance for mother–child health [1–7]. The wide variety of processes in which iron is involved means that prenatal iron deficiency might negatively affect the child’s development and, especially, brain functioning [8–10]. However, current evidence has mostly originated from animal studies and observational studies in humans. Only a few randomized controlled trials (RCT) have been conducted to evaluate the association between maternal iron status and the child’s cognitive and motor performance. Nevertheless, some important findings have arisen from these studies. In fact, since the harmful effects of prenatal suboptimal iron status have been observed in the short term [11], they could also persist even after correcting iron deficit [12–18]. Thus, iron supplements are usually prescribed to pregnant women [19–21], with good results in improving the serum iron-related biomarkers during gestation, [22–25] although they have not been always associated with better developmental outcomes for their children [26–28]. Also, it should be pointed out the risk of iron overload when an iron-replete woman or those with mutations in the *HFE* gene –which increases the intestinal iron absorption, especially in homozygotes but also in heterozygosis to a lesser extent– receives routine prenatal iron supplements [29–31], as well as the negative consequences that have also been associated with prenatal excess iron on the neuropsychological functions of the child [14, 22, 25, 32, 33]. In this regard, it is important to highlight that the overall prevalence of *HFE* gene alterations in our population is quite high [34]. After some authors have concluded that both iron deficit and excess may injure the child’s cognitive and motor development [32, 35, 36], they advise that prenatal iron supplementation should be individualized considering maternal iron stores, as well as other lifestyle and biological conditions [37, 38]. We supported this advice after having shown by a community-based Randomized Clinical Trial (RCT) the effectiveness of adapting iron doses to maternal haemoglobin (Hb) levels early in pregnancy in preventing iron deficiency, anaemia, and haemoconcentration [39]. [34, 40]In this study, the obtained results were adjusted for iron stores and specific *HFE* genotypes, among other women’s characteristics, demonstrating that Hb levels in early pregnancy (at gestational week 12) were related to serum ferritin concentrations and abnormalities in the *HFE* gene. Like ours, some authors advocated personalizing iron supplements used during pregnancy to provide the most appropriate supply of iron for each woman, which helps to prevent iron deficiency in some women and iron excess in others, improving the maternal iron status during pregnancy in all of them [22, 23, 25, 41]. However, little research has focused on the effects of this prenatal iron supplementation on the child’s neurocognitive abilities, and the available studies show inconsistent results [33, 42–44].

We have already shown in our study population that adapting prenatal iron supplementation (80, 40, and 20 mg) to early pregnancy iron levels (normal, Hb 110–130 g/L, or normal-high Hb > 130 g/L) in non-anaemic women, prevents iron imbalances during pregnancy, both due to iron deficiency and excess [39]. Specifically, we found that in women at risk of iron deficiency (those with normal Hb levels at the beginning of pregnancy), a daily dose of 80 mg of iron reduced the risk of iron deficiency, compared to women in the control group receiving 40 mg iron daily, without increasing the risk of excess of iron that can be caused by supplementation with high doses. Similarly, in women at risk of haemoconcentration (those with basal normal-high Hb levels), the tested iron dose of 20 mg daily prevented haemoconcentration compared to the control group, without causing iron deficiency. As a step forward, the present study aimed to evaluate the association between different prenatal doses of iron and the child’s neurodevelopment under the hypothesis that having corrected the women’s iron status by adapting prenatal iron supplementation to their individual needs would also have beneficial effects on the child’s neurodevelopment.

## Methods

### Study design and data collection

**Fig. 1:**
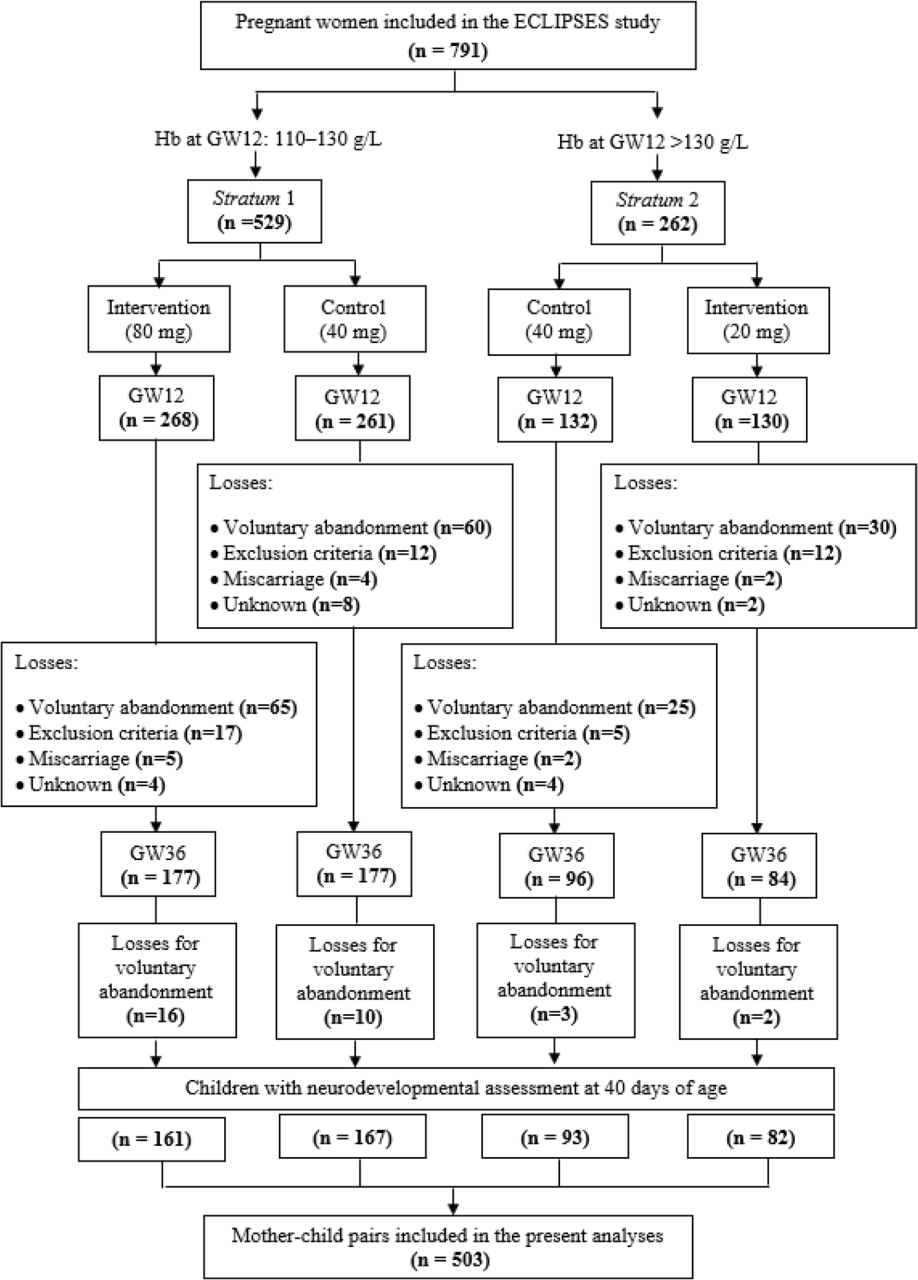
Flowchart of the study. Hb, haemoglobin; GW, gestational week

The present work is the follow-up of the ECLIPSES study, a community-based RCT that aimed to assess the effectiveness of different doses of prenatal iron supplementation on the maternal iron status at the end of gestation, and now, on the child’s neurodevelopment. The ECLIPSES study was conducted in the province of Tarragona (Catalonia, Spain) on women recruited before gestational week 12 between 2013–2017 and allocated into two groups according to their Hb levels aiming to prevent an iron deficit in women from the *Stratum* 1 (initial Hb = 110–130 g/L) and risk of developing iron excess in those from the *Stratum* 2 (initial Hb > 130 g/L). We used Hb levels as a screening biomarker because they are usually related to iron stores and *HFE* gene mutations [34, 40]. Women in *Stratum* 1 were randomly prescribed a daily dose of 40 or 80 mg of iron supplements, while those in *Stratum* 2 received 40 or 20 mg of iron daily (Fig. 1). Since previous literature has already made it clear that prenatal iron supplementation is recommended and leads to better pregnancy outcomes than non-supplementation, we did not include a control group of non-supplemented women. Otherwise, women in each *Strata* receiving 40 mg/day, which is the commonly prescribed dose of iron, were considered the control group against which the higher and lower iron dose interventions were performed. The prescription was triple-blinded, so neither the supplement providers, the health workers, nor the researchers knew what dose of iron each woman received until the end of the study. The women were instructed to take one pill a day. At the next study visit, they were to return any leftover pills to assess compliance. This was done by comparing the number of leftover pills with the participants' self-reported compliance. Compliance was considered good when women had forgotten to take the supplement less than twice a week while adherence was considered low when they had forgotten two or more times a week at any of the study visits.

The ECLIPSES study was registered at www.clinicaltrialsregister.eu as EudraCT number 2012–005,480-28 and its methodological details can be found extended elsewhere [39, 45].

Women were visited in the first, second, and third trimesters of pregnancy, and on average at 40 days post-partum. A summary of maternal information recorded by midwives from questionnaires was as follows:

- Clinical and obstetrical history: maternal age, parity, pregnancy planning.
- Anthropometric measurements: weight and height. Body mass index (BMI, kg/m^2^) was calculated.
- Dietary assessment: self-administered food frequency questionnaire (FFQ) previously validated in our population [46]. Participants reported usual food consumption retrospectively at weeks 12, 24, and 36 of pregnancy and 40 days post-partum. The FFQ consisted of 45 items classified into 12 food groups: 1–read and processed meat, 2.–poultry, fish, and eggs, 3.–fruits, 4.–vegetables, 5.–dairy products, 6.–salted cereals (breakfast cereals, bread, pasta, and rice), 7.–sweet cereals (biscuits, pastries), 8.–legumes, 9.–nuts, 10.–sweets, 11.–sweetened beverages, 12.–alcoholic drinks. The FFQ data were reviewed and analysed by trained nutritionists, who calculated the intake of each food group in grams/day. Additionally, the women’s degree of adherence to the Mediterranean diet was calculated using an rMED score based on the intake of 9 components of this diet. Each rMED component (apart from alcohol) was expressed in grams per 1000 kcal/day (to express intake as energy density) and was divided by terciles of dietary intake. Each tercile was assigned a value of 0, 1, and 2 points. Out of the 9 components of the rMED, 6 of them (fruit, vegetables, legumes, cereals, fresh fish and seafood, and olive oil) scored positively, while 2 scored negatively (total and processed meat, dairy products). Alcohol was scored as a dichotomous variable (0 for women who consumed alcohol, and 2 for women who did not drink alcohol). The score assigned to each pregnant woman thus ranged from 0 points indicating minimum adherence to 18 points indicating maximum adherence to the Mediterranean diet. The total rMED score was classified into three categories: 0–6 it was considered as ‘‘low’’, 7–10 as ‘‘medium’’, and 11–18 as ‘‘high’’. Extended information can be found in Jardí et al. [47].
- Lifestyle at the time of recruitment: use of prenatal supplements other than iron, smoking habit (using the Fagerström test [48]), and physical activity (using the short form of the International Physical Activity Questionnaire [IPAQ] [49]). The IPAQ assesses physical activity considering the following domains: leisure time, domestic activities, work-related physical activity, and transport-related physical activity. Within these domains, the IPAQ short form focus on walking, moderate-intensity activities, and vigorous-intensity activities. Total scores are computed based on the duration (in minutes) and frequency (in days) of each type of activity. The IPAQ offers the specific algorithms to obtain the classification of “low”, “moderate”, and “high” physical activity by combining the duration and frequency of different types of activities.Sociodemographic characteristics: ethnic origin, familiar socioeconomic status (SES) calculated from educational level and occupational status both from participants and their partners.
- Maternal anxiety status: State-Trait Anxiety Inventory (STAI) [50]. The STAI test assessed two separate concepts of anxiety, each with 20 items. On one hand, anxiety as a state assesses a transient emotional state, characterised by subjective, consciously perceived feelings of alertness and apprehension and by hyperactivity of the autonomic nervous system. On the other hand, anxiety as a trait indicates a relatively stable anxious propensity that characterises individuals with a tendency to perceive situations as threatening.Post-partum depression: Edinburgh Postnatal Depression Scale (EPDS) [51].

Blood samples were collected in 2 tubes of 7.5 ml, one containing EDTA as anticoagulant and the other without anticoagulant. The samples were transported to the BioBank for immediate analyses. Before processing, the samples in the EDTA tubes were inversion-mixed 10 times to ensure that the blood was mixed, then centrifuged at 4°C to separate plasma. The tube without anticoagulant was left without mixing for 30 minutes at room temperature to enable coagulation, then the serum was separated also by centrifugation. All the samples were stored at -80°C. DNA was extracted and stored as well at -80°C for subsequent genetic analyses. The stored samples in the BioBank were thawed at the end of the clinical study and processed simultaneously to minimize inter-batch variation. Biochemical determinations of Hb, serum ferritin (SF) and cortisol were done by immunochemiluminescence at each trimester of pregnancy. The serum concentration of C-reactive protein (CRP) was measured by immunoturbidimetry also at each trimester of pregnancy. Plasma polyunsaturated fatty acids (PUFA) concentrations were analysed by using a combination of gas chromatography–mass spectrometry (GC-MS) after their derivatization to methyl ester (FAMEs) due to their higher volatility. [52] Detailed information on laboratory procedures for PUFA measurements can be found in Aparicio et al. [53] Serum concentrations of vitamin D were quantified by an automated chemiluminescent immunoassay method as described in Díaz-López et al. [54] The rationale for including serum concentrations of vitamin D and fatty acids in the analyses is that these components are involved in brain development, so maternal levels during pregnancy may influence foetal neurodevelopment. [55–57] Folate and vitamin B_12_ measurements were also done by using a chemiluminescence immunoassay only in the first trimester of gestation. Then, RBC folate concentration was calculated as follows: (serum folate in haemolysed whole blood * dilution factor in haemolysis * 100)/haematocrit. [58] Genetic analyses to detect mutations in the *HFE* gene were done using polymerase chain reaction (PCR) and digestion with specific enzymes.

As for the infants’ information, the following data were recorded at birth: sex, gestational age (calculated based on the time elapsed since the first day of the last self-reported menstrual period), Apgar test score, type of feeding, and anthropometric measurements including length, weight, and head circumference. At 40 days of age, children were visited again and information about the type of feeding as well as weight and height measurements were recorded that time.

### Outcome

The individualized assessment of the child neurodevelopment was performed by two trained psychologists in the facilities of the health care centre participating in the study at the average age of 40 days using The Bayley Scales of Infant Development, 3^rd^ edition (BSID-III) [59]. This test consists of three general scales (cognitive, language, and motor) obtaining a standardized IQ score (mean of 100 and a standard deviation of 15) and four subscales (expressive language, receptive language, fine motor, and gross motor) obtaining a standardized scalar score (mean of 10 and a standard deviation of 3). Higher scores represent better development.Continuous BSID-III scores for general scales were categorized as follows according to the test rates: low (scores < 85), middle (scores ≥ 85–115), and high (scores ≥ 115). Similarly, the classification was as follows for subscales: low (scores < 7), middle (scores ≥ 7–13), and high (scores ≥ 13). Given the low number of children in the “high” category in cognitive and language scales, they were merged with those in the “middle” category when performing logistic regression analysis. In the case of the motor scale, children in the “low” category were merged with those in the “middle” category for the same reason.

### Statistical analyses

The analyses were considered as *per protocol* analyses and were stratified according to the design by Hb concentration category at baseline.. Bivariate analyses to describe the variables of interest were performed using the conventional statistical techniques: Student T and ANOVA tests for continuous variables using mean and standard deviation (SD) and Chi-square test of percentages for categorical ones. Natural logarithm (Ln) transformation was applied to normalize the distribution of SF, increasing the validity of analyses.

Linear and logistic regression models were used to assess the effect of different doses of prenatal iron supplementation (S*tratum* 1: 80 mg vs control, *Stratum* 2: 20 mg vs control) on the child’s neurodevelopment. Crude and adjusted estimates are shown. Based on previous knowledge, the models were adjusted for those maternal and child variables that could affect the studied relationship as follows: maternal age at recruitment, parity (yes or no), pregnancy planning (yes or no), familiar socioeconomic status (low, middle, high), smoking at recruitment (yes or no), baseline maternal BMI (normal weight, overweight, obesity), gestational weight gain, maternal anxiety during pregnancy, post-partum depression, serum levels of Hb, ferritin, vitamin D and polyunsaturated fatty acids at the first and third trimester of pregnancy, serum levels of RBC folate and vitamin B_12_ at the first trimester of pregnancy, physical activity during pregnancy (low, moderate, high), adherence to the Mediterranean diet (low, middle, high) and daily energy intake at the first trimester of pregnancy, child’s age at assessment, sex, gestational age, Apgar test scores (< 7 or ≥ 7 points), and head circumference at birth.

The statistical analyses were done using the SPSS software (version 27.0 for Windows; SPSS Inc., Chicago, IL, USA).

### Ethical approval

The study was designed in agreement with the Declaration of Helsinki/Tokyo. All procedures involving human subjects were approved by the Clinical Research Ethics Committee of the Jordi Gol University Institute for Primary Care Research [Institut d’Investigació en Atenció Primària; IDIAP], the Pere Virgili Health Research Institute [Institut d’Investigació Sanitària Pere Virgili; IISPV] and of the Spanish Agency for Medicines and Medical Devices [Agencia Española del Medicamento y Productos Sanitarios; AEMPS]. Signed informed consent was obtained from all women participating in the study.

The quality of the present community-based RCT has been assessed by the Consolidated Standards of Reporting Trials (CONSORT).

## Results

The current analyses consisted of 503 mother–child pairs from the ECLIPSES study, from which data on child neurodevelopment assessment at 40 days of age was available. Table 1 summarizes the maternal baseline characteristics, being remarkable that the median ± interquartile range maternal age was 31 ± 7 years, that near of 40% were overweight or obese 14.7% were smokers at the conceptional time, and most of them were White, had low or middle SES, and had planned the pregnancy. We also found that more than half of women (61.2%) showed low-middle adherence to the Mediterranean diet and moderate physical activity during pregnancy (66.9%). There was a high compliance to the intervention throughout pregnancy (around 94%). No association was found regarding sociodemographic characteristics and lifestyle between participants whose data were included or not included in the present analyses (Supplementary Table 1).

**Table 1:** Maternal characteristics

	***Stratum* 1 (Hb 110–130 g/L)**		***Stratum* 2 (Hb > 130 g/L)**	
	**Intervention (80 mg/d)** **(*n* = 161)**	**Control** **(40 mg/d)** **(*n* = 167)**	**Intervention (20 mg/d)** **(*n* = 82)**	**Control** **(40 mg/d)** **(*n* = 93)**
**Baseline**				
Age, years	31 ± 7	31 ± 7	31 ± 7	32 ± 7
Parity, yes	55.9 [90]	58.7 [98]	47.6 [39]	54.8 [51]
Pregnancy planning, yes	78.9 [127]	81.4 [136]	86.6 [71]	80.6 [75]
Body mass index				
Underweight	1.2 [2]	1.2 [2]	2.4 [2]	2.2 [2]
Normal weight	57.8 [93]	64.1 [107]	62.2 [51]	50.5 [47]
Overweight	28.6 [46]	21.6 [36]	19.5 [16]	33.3 [31]
Obesity	12.4 [20]	13.2 [22]	15.9 [13]	14.0 [13]
Smoking, yes	15.6 [25]	12.1 [21]	15.9 [13]	15.1 [14]
Familiar socioeconomical status^a^				
High	16.8 [27]	22.2 [37]	15.9 [13]	20.4 [19]
Middle	65.8 [106]	67.7 [113]	68.3 [56]	71.0 [66]
Low	17.4 [28]	10.2 [17]	15.9 [13]	8.6 [8]
Ethnicity				
White	79.5 [128]	79.6 [133]	86.6 [71]	69.9 [65]
Asian	0.6 [1]	0 [0]	0 [0]	2.2 [2]
Black	2.5 [4]	3.0 [5]	1.2 [1]	0.0 [0]
Arab	6.8 [11]	4.2 [7]	2.4 [2]	8.6 [8]
Latin American	8.7 [14]	10.8 [18]	8.5 [7]	12.9 [12]
**Whole pregnancy**				
Adherence to the Mediterranean diet^a^				
Low-Middle	64.0 [103]	59.9 [100]	69.5 [57]	67.7 [63]
High	36.0 [58]	40.1 [67]	30.5 [25]	32.3 [30]
Physical activity^a^				
Low	14.9 [24]	18.0 [30]	18.3 [15]	26.9 [25]
Moderate	55.3 [89]	52.1 [87]	45.1 [37]	36.6 [34]
High	18.0 [29]	12.6 [21]	17.1 [14]	24.7 [23]
Anxiety assessment^b^				
Trait	18.04 (9.30)	15.19 (8.06)	16.82 (9.85)	14.43 (8.03)
State	18.07 (7.77)	15.41 (6.79)	16.99 (8.03)	15.84 (7.21)
**After delivery**				
Post-partum depression^**c**^	7.58 (5.22)	6.67 (4.78)	6.50 (5.46)	6.44 (4.36)

Data are expressed in mean (SD) for continuous normally distributed variables, median ± interquartile range for continuous non-normally distributed variables, and % [n] for categorical variables

^a^For an explanation of how categories were defined see the Methods section

^b^Measured by STAI questionnaire (score range: 0 to 60 points). Trait means a relatively stable, anxious propensity that characterises individuals with a tendency to perceive situations as threatening. State means a transient emotional state, characterised by subjective, consciously perceived feelings of attention and apprehension and by hyperactivity of the autonomic nervous system

^**c**^Measured by Edinburg questionnaire (score range: 0 to 30 points)

Maternal concentrations of iron-related biomarkers in the first and third trimesters are shown in Supplementary Table 2. As for the maternal iron status, since anaemia was an exclusion criterion for the recruitment and haemoconcentration is a condition associated with late pregnancy, only data for the third trimester of gestation are shown. The participants showed a low prevalence of iron-deficiency anaemia in all the iron groups (1 to 5%), without any association among them. As for haemoconcentration, the overall prevalence was 13.7% with the higher percentage being shown by women in *Stratum* 2. Additional information on maternal levels of vitamin B_12_ and RBC folate at the beginning of pregnancy are also shown in Supplementary Table 2.

The child’s characteristics according to the intervention group are depicted in Table 2. The results showed that 49.5% of the participating children were girls, had a mean gestational age of 39.7 weeks, and a median age of 47 ± 14 days when neurodevelopment was assessed. Regarding the BSID-III scores, they were normally distributed in our study population and the mean scores obtained for all the children were in the normal ranges at each scale when they were analysed as a continuous variable. However, when the scores were categorized considering the clinical cut-off point for normality (85 or 7 points for main scales and subscales, respectively), a small percentage of children were under the normality for language development (8.1%, *n* = 45) and, specifically, for expressive language development (11.9%, *n* = 65). No association was found in the BSID-III scores or in the percentage of children below the cut-off point considered normal, between the different doses of iron in any *Strata*.

**Table 2:** Characteristics of children according to the dose of maternal iron supplementation during pregnancy

	***Stratum* 1 (Hb 110–130 g/L)**		***Stratum* 2 (Hb > 130 g/L)**	
	**Intervention (80 mg/d)** **(*n* = 161)**	**Control** **(40 mg/d)** **(*n* = 167)**	**Intervention** **(20 mg/d)** **(*n* = 82)**	**Control** **(40 mg/d)** **(*n* = 93)**
Age at assessment, days	47 ± 15	47 ± 13	47 ± 14	47 ± 14
Sex, girl	51.1 [81]	51.6 [83]	45.9 [37]	45.5 [44]
Gestational age, weeks	39.62 (1.51)	39.74 (1.38)	39.61 (1.50)	39.85 (1.30)
Apgar test score ≥ 7 points, %	98.8 [159]	100 [167]	100 [82]	98.9 [92]
Breastfeeding, yes				
At birth	63.9 [98]	68.3 [114]	63.1 [50]	63.6 [60]
At assessment	54.4 [87]	63.5 [106]	51.2 [41]	56.5 [52]
**Neurodevelopment**				
Cognitive development^a^	100.81 (8.86)	101.79 (8.55)	101.28 (9.16)	103.28 (8.95)
Score < 85, %	3.1 [5]	2.4 [4]	3.7 [3]	1.1 [1]
Language development^a^	96.44 (8.34)	95.92 (8.61)	95.04 (7.35)	97.34 (8.23)
Score < 85, %	8.0 [13]	10.1 [17]	8.5 [7]	4.3 [4]
*Expressive language*^b^	8.04 (1.39)	8.16 (1.65)	7.88 (1.41)	8.09 (1.78)
Score < 7, %	9.3 [14]	12.5 [20]	11.0 [9]	16.1 [15]
*Receptive language*^b^	10.71 (2.12)	10.41 (2.17)	10.43 (2.04)	10.98 (2.04)
Score < 7, %	4.3 [7]	4.2 [7]	2.4 [2]	4.3 [4]
Motor development^a^	107.27 (12.74)	107.83 (10.27)	107.16 (10.02)	108.08 (11.35)
Score < 85, %	3.1 [5]	2.4 [4]	2.4 [2]	4.3 [4]
*Fine motor*^b^	11.46 (1.98)	11.53 (1.85)	11.39 (1.92)	11.43 (2.09)
Score < 7, %	0.6 [1]	0.6 [1]	1.2 [1]	2.2 [2]
*Gross motor*^b^	11.12 (2.34)	11.00 (2.26)	11.01 (2.34)	11.19 (2.46)
Score < 7, %	0 [0]	0.6 [1]	0 [0]	0 [0]

Data are expressed in median ± interquartile range, mean (SD) and % [n]

^a^The normal score range for BSID-III was 85–115

^b^The normal score range for BSID-III was 7–13

**Fig. 2:**
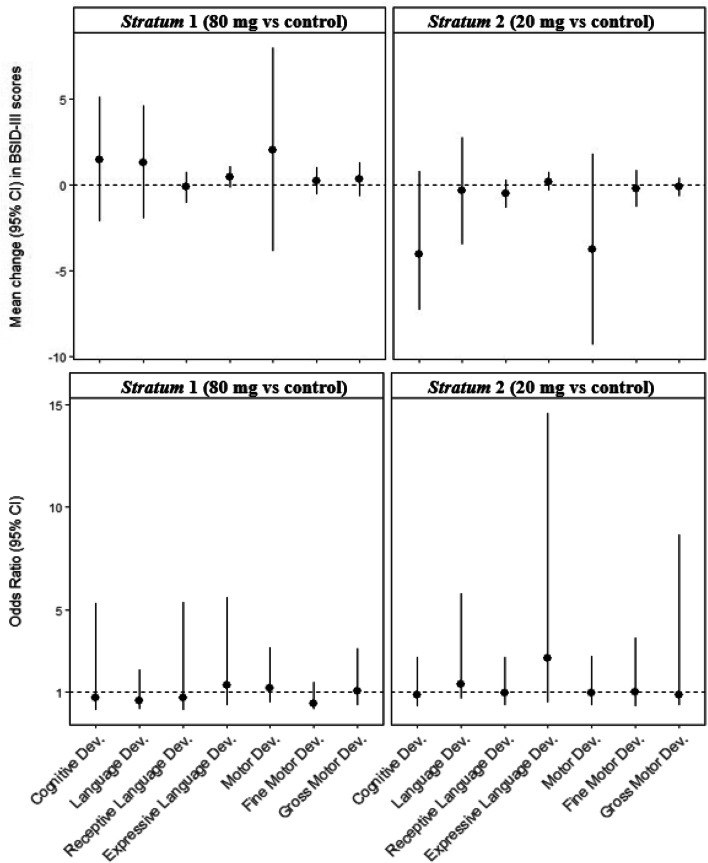
Effect of iron supplementation on the neurodevelopment of children at around 40 days of life. The control group in each *Stratum* were women who received the commonly prescribed dose of 40 mg/d of iron. Models adjusted for maternal age at recruitment, parity, pregnancy planning, familiar socioeconomic status, smoking at recruitment, baseline maternal body mass index, gestational weight gain, maternal anxiety during pregnancy, postpartum depression, serum biomarker levels at the first and third trimester of pregnancy (haemoglobin, ferritin, vitamin D and polyunsaturated fatty acids), serum biomarker levels at the first trimester of pregnancy (red blood cell folate and vitamin B_12)_, physical activity during pregnancy, adherence to Mediterranean diet and daily energy intake at the first trimester of pregnancy, child’s age at assessment, child’s sex, gestational age, Apgar test scores, head circumference at birth, and type of feeding at birth and assessment. Odds Ratios express the chance to go from low to middle-high cognitive development and language development, and from low-middle to high motor development

In multiple linear regression analysis, no association was found between prenatally prescribed iron doses in *Stratum* 1 or 2 about BSID-III scores on any of the developmental scales in children at 40 days of age, including cognitive, language, and motor development (Table 3, Fig. 2). Neither association was found between the doses of iron in any of the groups in the logistic regression analysis on the chances of moving from low to medium–high mental and language development, and from low-medium to high motor development (Table 3, Fig. 2).

**Table 3:** Effect of iron supplementation on the neurodevelopment of children at around 40 days of life

	***Stratum* 1** **(0: 80 mg/d, 1: 40 mg/d)**		***Stratum* 2** **(0: 40 mg/d, 1: 20 mg/d)**	
	**β (95%CI)**	**OR (95%CI) **^**a**^	**β (95%CI)**	**OR (95%CI) **^**a**^
**Cognitive development**				
Crude model	0.98 (-0.82, 2.87)	1.31 (0.34, 4.95)	-2.00 (-4.71, 0.70)	0.29 (0.03, 2.81)
Adjusted model	1.46 (-2.15, 5.07)	0.68 (0.09, 5.29)	-4.04 (-7.27, 0.80)	0.85 (0.26, 2.68)
**Language development**				
Crude model	-0.53 (-2.37, 1.32)	0.78 (0.36, 1.65)	-2.31 (-4.65, 0.03)	0.48 (0.14, 1.71)
Adjusted model	1.30 (-1.99, 4.59)	0.54 (0.14, 2.05)	-0.36 (-3.47, 2.75)	1.35 (0.65, 5.79)
Receptive language				
Crude model	0.30 (-0.77, 0.17)	1.04 (0.36, 3.03)	-0.55 (-1.16, 0.06)	1.80 (0.32, 10.08)
Adjusted model	-0.14 (-1.04, 0.75)	0.72 (0.10, 5.35)	-0.52 (-1.32, 0.28)	0.94 (0.32, 2.68)
Expressive language				
Crude model	0.12 (-0.22, 0.45)	0.70 (0.34, 1.44)	-0.21 (-0.69, 0.27)	1.56 (0.64, 3.78)
Adjusted model	0.44 (-0.19, 1.06)	1.30 (0.30, 5.61)	0.18 (-0.36, 0.72)	2.64 (0.48, 14.58)
**Motor development**				
Crude model	0.56 (-1.96, 3.08)	1.03 (0.60, 1.78)	-0.92 (-4.13, 2.30)	0.68 (0.33, 1.42)
Adjusted model	2.04 (-3.88, 7.96)	1.19 (0.45, 3.15)	-3.76 (-9.30, 1.78)	0.91 (0.30, 2.73)
Fine motor				
Crude model	0.07 (-0.34, 0.49)	0.78 (0.41, 1.47)	-0.04 (-0.64, 0.56)	1.16 (0.47, 2.82)
Adjusted model	0.23 (-0.58, 1.03)	0.43 (0.13, 1.43)	-0.24 (-1.29, 0.81)	0.97 (0.26, 3.61)
Gross motor				
Crude model	-0.12 (-0.62, 0.38)	0.73 (0.40, 1.32)	-0.18 (-0.90, 0.53)	0.71 (0.32, 1.59)
Adjusted model	0.32 (-0.65, 1.30)	1.03 (0.34, 3.11)	-0.13 (-0.66, 0.41)	0.84 (0.31, 8.65)

Doses of iron: *Stratum* 1 (80 vs 40 mg/d) and *Stratum* 2 (40 vs 20 mg/d)

Models adjusted for maternal age at recruitment, parity, pregnancy planning, familiar socioeconomic status, smoking at recruitment, baseline maternal body mass index, gestational weight gain, maternal anxiety during pregnancy, postpartum depression, serum biomarker levels at the first and third trimester of pregnancy (haemoglobin, ferritin, vitamin D and polyunsaturated fatty acids), serum biomarker levels at the first trimester of pregnancy (red blood cell folate and vitamin B_12)_, physical activity during pregnancy, adherence to Mediterranean diet and daily energy intake at the first trimester of pregnancy, child’s age at assessment, child’s sex, gestational age, Apgar test scores, head circumference at birth, and type of feeding at birth and assessment

^a^Odds ratios express the chance to go from low to middle-high cognitive development and language development, and from low-middle to high motor development

## Discussion

The need for preventive prenatal iron supplementation to avoid iron deficiency is well known, although favourable results on the child’s development have not always been observed. Routine preventive supplementation with standard doses of iron for all women could probably be insufficient for those who start pregnancy with low iron levels and too much for those with high initial iron concentrations. Thus, iron supplementation adapted to maternal needs would prevent both extremes and, in turn, improve child outcomes. This community-based study found that adapting prenatal iron supplementation in non-anaemic women to their individual needs to prevent iron deficit and excess, led to similar results in their children's cognitive, language, and motor development in all iron supplementation groups (daily 20 mg, 80 mg, and control group of 40 mg), resulting in a lack of association between the tested doses and the control group in each *Strata* for child’s neurodevelopment.

Little research assessing the effect of prenatal iron supplementation on the child’s neurodevelopment has been conducted on well-nourished non-anaemic women, and studies until now only evaluated the effect of taking or not prenatal iron supplements, without considering different doses according to the individual needs of each woman. Results from the AMBIT study [26, 27], conducted in non-anaemic Australian pregnant, indicated no effects of prenatal iron supplementation (20 mg daily) compared to placebo on the offspring’s intelligence and behavioural skills at 4 and 6–8 years of age. Some years later, a study conducted in 9-months-old Chinese children assessing the effectiveness of antenatal (60 mg daily vs placebo) and infant iron supplementation found an improvement in the child’s gross motor development, although it was not due to iron supplementation during pregnancy, but during infancy [28]. Despite the tested doses in these studies being around the usual doses in routine supplementation, considering that the participating women were not anaemic, they could have been inadequate depending on their iron stores and other factors. In this regard, women with homozygous mutations in the *HFE* gene, which increase iron absorption, may be at risk of iron overload if they receive iron supplements [29–31]. Despite the low prevalence of homozygosis for *HFE* gene mutations in our study population (only 4.8% for H63D genotype), the prevalence of having any *HFE* gene mutation is around 46% in the Mediterranean population [34, 60], which turn it into an important risk factor to consider when prescribing iron supplementation. There are some indications from observational studies that not only iron deficiency but also iron excess negatively affects the child’s neurodevelopment [18, 32, 33, 35, 36] and, based on that, some main researchers in this field have proposed to adapt the prenatal iron supplementation to the individual’s requirements to mitigate the potential damage from any maternal iron imbalance [22, 23, 25, 41]. However, few studies have tested this hypothesis, obtaining inconsistent results [37, 61]. We observed that our intervention successfully corrected maternal iron status when compared with the estimates of the prevalence of anaemia and haemoconcentration during pregnancy. While the prevalence of anaemia in Europe is around 25% in pregnant women [19, 62, 63], we found that only 3.3–5% of participants at risk developed it at the end of pregnancy. On the other hand, despite the estimates indicating that up to 42% of women suffer from haemoconcentration in industrialized countries [25, 64], we observed a prevalence of 15.6–25.6% among participants at risk of iron excess. We believe, therefore, that having provided the most appropriate amount of iron for each woman helping them to reach an optimal iron status in most cases has been the physiological mechanism underlying the lack of a remarkable association between different prenatal iron doses and child’s neurodevelopment. We found that only 2.6%, 8.1%, and 3% of children obtained scores below the normal range for cognitive, language, and motor development scales, respectively. The high heterogeneity among the epidemiological studies assessing the effect of prenatal iron supplementation on the child’s neurodevelopment makes it difficult to compare our findings. Nonetheless, some evidence from observational studies indicate that failure to prevent women from suffering from both iron deficiency and excess in pregnancy results in neurodevelopmental impairment in children. That is the case of two Spanish studies [11, 18] and others from Vietnam [65] and China [66] from which the authors concluded that iron deficiency or anaemia in late pregnancy, compared with having a correct iron status, may be associated with lower motor scores in young children. Similarly, maternal iron deficiency during pregnancy can result in poorer cognitive and language abilities in children, according to some of those studies [18, 67]. But, as previously discussed, that prenatal iron excess could entails harmful consequences for child neurodevelopment has also been stated in the literature, especially associated with cognitive function in this case [32, 36, 68]. And, going further, a couple of studies made sense of the present work, showing in their study population that the association between maternal iron status and child neurodevelopment is sometimes inverted U-shaped [69, 70]. Considering the available evidence, our results suggest that preventive prenatal supplementation with different doses of iron as long as they are in a range appropriate to each woman's needs, i.e. adapted to their initial Hb levels, lead to similar neurodevelopmental outcomes in infants at birth. The main strengths of the present work were the study design, which was a community-based triple-blinded community-based RCT and the extensive data collection regarding sociodemographic conditions, clinical and lifestyle information from both mothers and children. Also, It should be noted that women in our study were non-anaemic when they were recruited in the first trimester of pregnancy and started iron supplementation. This does not mean that they cannot become anaemic during pregnancy. However, by adjusting for maternal serum ferritin and Hb concentrations in both the first and third trimesters of pregnancy, we were able to rule out the possible effect of maternal iron status and estimate the true effect of prenatal iron supplementation on the child’s neurodevelopment. However, some limitations should also be considered. First, despite being common in community-based intervention studies implying several visits and a long follow-up, the substantial drop-out that occurred could have reduced the statistical precision and may have led to biased estimates of intervention effects. Second, although the BSID-III is an internationally used and recognised tool for assessing the child’s cognitive function, the neurodevelopmental assessment shows low stability in early childhood [71], which could have led to estimates of nullity. And finally, residual confounding, due to unmeasured or unknown risk factors that may occur even after adjustment for known potential confounders, could have been a limitation when interpreting our findings.

## Conclusions

Our findings suggest that in non-anaemic women at the start of pregnancy, there were no differences in the cognitive, language and motor development of children at 40 days of age between the dose of iron tested in each case (80 or 20 mg/d) –adjusted to initial Hb levels– compared to the dose of the control group (40 mg/d), which is the dose commonly used in clinical practice in SpainThe research in this regard is still scarce and further studies are guaranteed to better understand the possible effects of different types of prenatal iron supplementation on the child’s neurodevelopment, including the follow-up at older ages.

## Supplementary Information

**Additional file 1. Supplementary Table 1. **Maternal characteristics of participants included and non-included in theanalyses.

**Additional file 2. Supplementary Table 2. **Maternal concentrations of iron-related biomarkers, vitamin B12, and RBC folate.

## Data Availability

The datasets used and/or analysed during the current study are available from the corresponding author on reasonable request.

## Abbreviations

RCT: Randomized clinical trial
Hb: Haemoglobin
BMI: Body mass index
FFQ: Food frequency questionnaire
SES: Socioeconomic status
STAI: State-Trait Anxiety Inventory
EPDS: Edinburgh Postnatal Depression Scale
SF: Serum ferritin
RBC: Red blood cell
BSID: Bayley Scales of Infant Development
SD: Standard deviation
Ln: Natural logarithm
