# Supplementary material for: Adapting prenatal iron supplementation to maternal needs results in optimal child neurodevelopment: a follow-up of the ECLIPSES Study
Source: BMC Pregnancy Childbirth. 2022 Sep 17;22:710. doi: 10.1186/s12884-022-05033-y (PMC9482254; doi:10.1186/s12884-022-05033-y)
Supplement: Supplementary file 1 — Additional file 1. Supplementary Table 1. Maternal characteristics of participants included and non-included in theanalyses. [file 12884_2022_5033_MOESM1_ESM.docx]

| **Supplementary Table 1. Maternal characteristics of participants included and non-included in the analyses** | | | |
| --- | --- | --- | --- |
|  | **Included (n=503)** | **Non-included (n=288)** | **p value** |
| **Baseline** |  |  |  |
| Age, years | 31±7 | 29±7 | 0.894 |
| Parity, yes | 56.7 [285] | 50.1 [144] | 0.089 |
| Pregnancy planning, yes | 81.5 [410] | 77.7 [224] | 0.233 |
| Body mass index |  |  | 0.685 |
| Underweight | 1.4 [7] | 1.7 [5] |  |
| Normal weight | 58.9 [296] | 55.1 [159] |  |
| Overweight | 26.4 [133] | 27.5 [79] |  |
| Obesity | 13.3 [67] | 15.7 [45] |  |
| Smoking, yes | 14.7 [74] | 16.8 [48] | 0.198 |
| Familiar socioeconomic status |  |  | 0.098 |
| High | 14.9 [75] | 13.8 [40] |  |
| Middle | 41.4 [208] | 63.8 [183] |  |
| Low | 43.7 [220] | 22.4 [65] |  |
| Ethnicity |  |  | 0.852 |
| Caucasian | 81.1 [408] | 78.6 [226] |  |
| Asian | 0.6 [3] | 0 [0] |  |
| Black | 6.6 [3] | 2.1 [6] |  |
| Arab | 1.8 [9] | 8.4 [24] |  |
| Latin American | 9.9 [50] | 10.9 [31] |  |
| **Whole pregnancy** |  |  |  |
| Adherence to the Mediterranean diet |  |  | 0.107 |
| Low-Middle | 61.2 [308] | 64.3 [185] |  |
| High | 38.8 [195] | 35.7 [103] |  |
| Physical activity |  |  | 0.298 |
| Low | 21.4 [108] | 20.5 [59] |  |
| Moderate | 58.6 [294] | 64.7 [186] |  |
| High | 20.1 [101] | 14.8 [43] |  |
| Data are expressed in mean (SD) for continuous normally distributed variables, median ± interquartile range for continuous non-normally distributed variables, and % [n] for categorical variables. | | | |
