# Supplementary material for: Adapting prenatal iron supplementation to maternal needs results in optimal child neurodevelopment: a follow-up of the ECLIPSES Study
Source: BMC Pregnancy Childbirth. 2022 Sep 17;22:710. doi: 10.1186/s12884-022-05033-y (PMC9482254; doi:10.1186/s12884-022-05033-y)
Supplement: Supplementary file 2 — Additional file 2. Supplementary Table 2. Maternal concentrations of iron-related biomarkers, vitamin B12, and RBC folate. [file 12884_2022_5033_MOESM2_ESM.docx]

| **Supplementary Table 2. Maternal concentrations of iron-related biomarkers, vitamin B_12_, and RBC folate.** | | | | | | |
| --- | --- | --- | --- | --- | --- | --- |
|  |  | ***Stratum* 1 (Hb 110-130 g/L)** | |  | ***Stratum* 2 (Hb >130 g/L)** | |
|  |  | **80 mg/d**  **(n= 161)** | **40 mg/d**  **(n= 167)** |  | **40 mg/d**  **(n=93)** | **20 mg/d**  **(n=82)** |
| **First trimester** |  |  |  |  |  |  |
| Haemoglobin (g/L) |  | 123.55 (4.58) | 122.96 (5.28) |  | 135.86 (4.89) | 136.59 (4.72) |
| Serum ferritin (µg/L) |  | 38.9 ± 27.30 | 37.30 ± 23.30 |  | 41.80 ± 27.35 | 40.75 ± 31.05 |
| Vitamin B_12_ (µg/L) |  | 376.50 (112.51) | 370.24 (103.84) |  | 379.73 (154.82) | 365.83 (111.78) |
| RBC folate (nmol/L) |  | 581.17 (196.11) | 567.50 (162.57) |  | 545.34 (153.25) | 537.12 (145.44) |
| **Third trimester** |  |  |  |  |  |  |
| Haemoglobin (g/L) |  | 117.23 (8.44) | 117.53 (8.12) |  | 123.81 (10.02) | 120.47 (9.01) |
| Serum ferritin (µg/L) |  | 17.19 ± 11.25 | 16.00 ± 12.30 |  | 11.70 ± 8.10 | 10.85 ± 6.70 |
| Iron-deficiency anaemia^*^, % |  | 3.3 [5] | 5.0 [8] |  | 2.4 [2] | 1.0 [1] |
| Haemoconcentration, % |  | 7.7 [12] | 9.7 [16] |  | 15.6 [15] | 25.6 [21] |
| ^*^Iron-deficiency anaemia was defined as Hb<105 mg/L and SF<12 µg/L, and haemoconcentration as Hb>130 g/L. | | | | | | |
| RBC, red blood cell. | | | | | | |
